# A sub-Saharan African experience in the surgical management of soft tissue sarcomas in an oncology unit in: a retrospective cohort study

**DOI:** 10.11604/pamj.2019.33.207.15970

**Published:** 2019-07-15

**Authors:** Omobolaji Oladayo Ayandipo, Oludolapo Ola Afuwape, Oluwafunmilayo Yewande Soneye, Akintunde Taiwo Orunmuyi, Gbolahan Oladele Obajimi

**Affiliations:** 1 College of Medicine, University of Ibadan, Nigeria; 2 University College Hospital, Ibadan, Nigeria

**Keywords:** Soft tissue sarcomas, surgical management, sub-Saharan Africa

## Abstract

**Introduction:**

Soft tissue sarcomas (STS) consist of over 70 histologic subtypes and constitute only 1% of adult malignancies. The fulcrum of management is surgical resection with neoadjuvant or adjuvant treatment-chemoradiation.

**Methods:**

The study is a retrospective review of consecutive STS patients who had surgery at the University College Hospital, Ibadan, between October 2007-2017. Data extraction was from the admission and operative registers, theatre records and histology reports. Statistical analysis was done using the Statistical Package for Social Sciences (SPSS) version 20 (Chicago IL USA). Results were summarized as charts and graphs.

**Results:**

Five hundred and ninety six cases of STS were seen over the ten-year period. Of these, 383 (64.3%) patients had surgery and the case files of 326 (85.1%) of these patients was available for review. The duration of soft tissue swelling, ranged from 1-96 months. A third of the tumors were superficial while 68% were deep-seated. Oncoplastic reconstruction was done in 42(13%) patients. The resection margin was negative in 88%. A total of 202 patients were followed up regularly for between 24-36 months only.

**Conclusion:**

Patients who benefitted from definitive surgical treatment for STS were found to be the young and middle age group. These patients had extended duration of symptoms with lesions > 5cm in size. Truncal and visceral STS had the worst prognosis. A Multi-Disciplinary Tumor (MDT) board for STS and a robust follow up would enhance the management of STS in a low resource setting.

## Introduction

Soft tissue sarcomas (STS) represents the uncommon, but ubiquitous group of neoplasms of mesenchymal tissue which although apt to be found in most organ systems are all the same rare in overall prevalence. They constitute only one percent (1%) of all adult malignancies [1-4]; but have over 70 histologic subtypes [5] with proven heterogeneity in terms of anatomic primary site, clinical presentation, prognosis and therapeutic features [6, 7]. Partly because of this rarity, management of soft tissue sarcomas remains complex and challenging and is dependent on various factors; such as tumor and treatment-related factors like the histologic subtype, tumor depth, grade, primary or recurrent, surgical margins and the need for neoadjuvant or adjuvant chemo-radiation [8-10]. The standard of care in managing STS is surgical resection with neoadjuvant or adjuvant chemo-radiation [11-14]. However, the main therapeutic burden is placed on surgery worldwide because of the need for local control via negative margins. The inadequacy of radiotherapy services in Low and Middle Income Country (LMIC) like Nigeria [15], the potential for toxicity to adjacent structure when employing radiotherapy and the unproven role of chemotherapy in managing different subtypes of STS all serve to magnify the importance of surgery [3, 16, 17]. The prognosis of patients with STS generally is poor when assessed over an extended period [18-20]. There are many publications on the outcome of surgical management of STS from developed countries [2, 6, 10] but few from developing ones. In this paper, we present our findings from a 10-year review of STS patients who had surgery in an academic surgical oncology practice in Ibadan, a University Teaching Hospital in Nigeria.

## Methods

We reviewed the case records of consecutive STS patients who had surgery at the UCH, Ibadan between October 2007 and October 2017. Data extraction was from the admission and operative registers, the theatre records, as well as the log of the histology reports. Only STS of connective, subcutaneous and muscular tissues were considered, with exclusion of bone-related sarcomas. The patients’ demographic data, clinico-pathologic profiles like primary or recurrent STS, anatomic site, tumor size, histologic types and grade, lymph node involvement, metastases, clinical stage, treatment outcome (margins, morbidity and mortality) and long-term outcomes were all recorded.

### Ethical issues

This study was conducted in compliance with the guidelines of the Helsinki declaration on biomedical research in human subjects. Confidentiality of the identity of the patients and personal health information was maintained. Individual patients consent was not required for this study since it is a retrospective study and a review of routine clinical activities. Approval for using the hospital records only for this study was obtained from the institution’s chairman, Medical Advisory Committee (CMAC), the chair of the hospital’s research and ethics committee before accessing the patients’ information at the medical records department.

### Clinical management of STS in our unit

A clinical review (history taking and thorough physical examination) preceded the diagnostic pathologic evaluation achieved by core-biopsy in cases presenting primarily to the division. Standard radiologic staging investigations included chest radiography, abdominopelvic ultrasound scanning, chest computed tomography (CT) and magnetic resonance imaging (MRI) of the anatomic region of the soft tissue swelling (if available and affordable). Following the decision to offer surgery, consideration for post-excision wound cover necessitated oncoplastic consult while those requiring neoadjuvant chemo-radiation were managed as such prior to surgery. The decision for adjuvant chemoradiation was taken after reviewing a combination of the clinical staging, intra-operative findings (tumor margins, resectability and relationship to contiguous structures) and the histology reports.

### Statistical analysis

All informations obtained were recorded in Microsoft Excel spreadsheet. Patients’ demography, clinico-pathologic profile, stage treatment offered as well as outcomes of treatment was summarized as percentages, proportions, mean and standard deviation. Statistical analysis was done using SPSS version 20 and the results were presented in charts and graphs.

## Results

**Figure 1:**
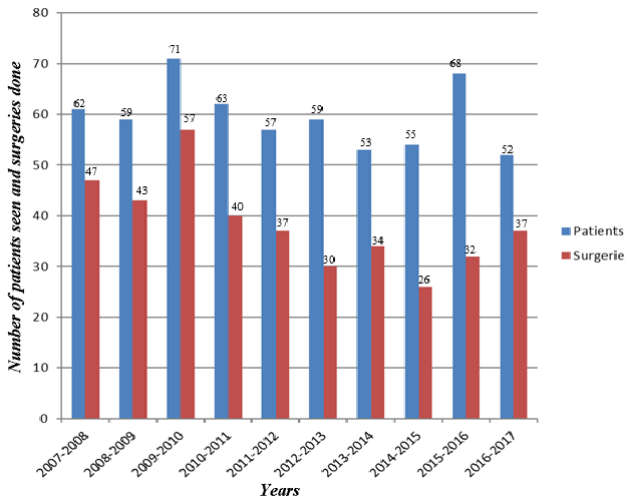
Showing the number of patients seen and the number of surgeries done in each year

We retrieved the records of 596 cases of STS over the ten-year period. Of these, 383 (64.3%) patients had surgery making an average of 38 oncologic excisions of STS per year. Figure 1 shows the number of cases diagnosed and those who had surgery within each year of the study. The case files of 326 patients constituting 85.1% of all surgeries were available for review. One hundred and sixty-seven (51.3%) were males and 159 (48.7%) were females. A total of 238 (73%) had primary disease, 88 (27%) were for local recurrence after initial surgical treatment at referring hospitals and 75 (23%) had metastasis to the lungs. The patients clinicopathologic and treatment characteristics are shown in Table 1. The duration of soft tissue swelling before presentation at the UCH, Ibadan ranged from 1 to 96 months with a mean (SD) of 13 (15.6) months. The swelling was described as painless in majority, 284 (87%) of the patients. The tumor size was less than 5cm in 59 (18%), between 5cm and 10cm in 94 (28.8%) and greater than 10cm in 173 (53.7%). Extremity lesions constituted 21.7%, while truncal, retroperitoneal, head and neck, visceral and other anatomical parts constituted 54.5%, 8.0%, 3.4%, 4.9%, 10.9% respectively.

**Table 1:** Patients clinico-pathologic characteristics

Characteristics	Number	Percentage of Total
**Age (years)**		
16-40	111	33.9
41-65	179	55.0
66 and above	36	11.1
Median (range)	47 (16-82)	
**Sex**		
Male	167	51.3
Female	159	48.7
**Primary presentation**		
No treatment /biopsy only	238	73.0
Prior excision	88	27.0
**Tumor location**		
Extremity	70	21.5
Trunk	167	51.2
Retroperitoneal	26	7.9
Head and neck	11	3.3
Visceral – gastrointestinal	16	4.9
Others	36	11.2
**Size (cm)**		
≤ 5	9	18.0
>5-10	94	28.8
>10	173	53.7
**Depth**		
Superficial	104	32.0
Deep	222	68.0
**Histologic grade**		
Low	95	29.1
Intermediate	55	17.0
High	176	53.9
**Histopathology**		
Liposarcoma	74	22.6
Malignant fibrous histiocytoma	36	11.0
Leiomyosarcoma	62	19.0
Fibrosarcoma	23	7.0
Synovial	16	5.0
Malignant peripheral nerve sheath	26	8.0
Tumor	12	3.9
GI stromal tumor	16	5.0
Embryonal-Rhabdomyosarcoma	13	4.0
Malignant-Phylloidestumor	48	14.5
**Others**		
**Overall margin status**		
Gross negative margins	287	88.0
Gross positive margins	39	12.0
Micro negative	264	81.0
Micro positive	62	19.0
**Chemotherapy (adjuvant)**		
Yes	65	19.9
No	261	80.1
**Radiotherapy**		
Yes	48	14.7
No	278	85.3

About a third (32%) of the tumors were adjudged superficial while 222 (68%) were deep-seated lesions. Histologically, they were low-grade neoplasms in 95 (29.1%), intermediate grade in 51 (17%) and high grade in 176 (53.9%). The three commonest histologic subtypes in descending order were liposarcoma (22.6%), leiomyosarcoma (19%) and malignant fibrous histiocytoma (11%). Neoadjuvant chemoradiation was administered in 57 (17.3%) while primary surgical excision was done in 269 (82.7%). Only 48 (14.7%) had adjuvant radiotherapy treatment and 65 (19.9%) had adjuvant chemotherapy. Oncoplastic reconstruction (split thickness skin grafts, local flaps) was done in 42 (13%). Some 7 limbs disarticulations were done at joint surgical resections with the orthopaedic team despite being of soft tissue origin. The tumor resection margin was grossly negative in 88% and positive in 12%; the histology was reported micro-negative in 81% and micro-positive in 19%. Thirteen percent of the positive margins were seen in the retroperitoneal and truncal STS. We found peritoneal sarcomatosis in 18 cases (5.5%) amongst the visceral group of STS, that is, the retroperitoneal and gastrointestinal stromal tumors (GIST).

**Figure 2:**
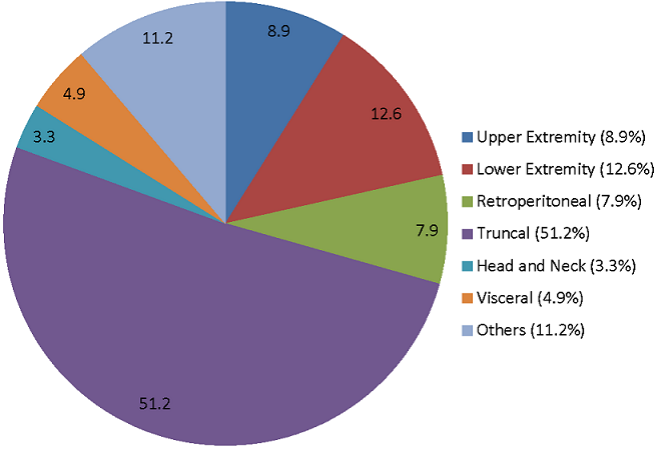
Anatomic location of the soft tissue sarcoma

**Figure 3:**
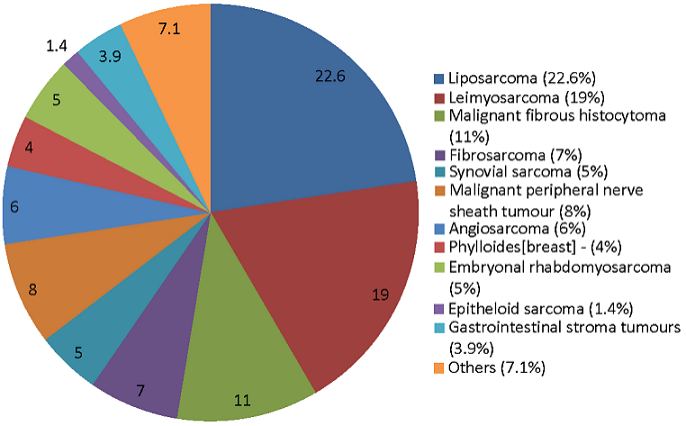
Histologic subtypes

Surgical drains were removed between the 4^th^ and 14^th^ post-operative day with a mean of 8 days in the proportion that needed a drain inserted. Drain removal was predicated on output being clear or straw colored and less than 50mls in the preceding 24 hours. Post-operative complications occurred in 61 (18.7%) with seroma, flap dehiscence, necrosis, wound infection being the commonest. The average length of hospital admission ranged from 5-15 days with a mean S.D of 11 days (SD). At least two-thirds, 238(67%) of the patients were available for as much as 15 months post hospital discharge for clinical evaluation in the surgical outpatient department (SOP). Two hundred and two (62%) were seen regularly for between 24-36 months; and then a decline in the follow-up rate ensued thereafter, such that by 36 months post-surgery most patients had dropped out of follow-up, except those that had local recurrence 56 (17%) and or metastatic disease 36 (11.0%) diagnosed (Figure 2, Figure 3).

## Discussion

In this review from the surgical oncology practice of a developing country, patients who benefitted from definitive surgical treatment for soft tissue sarcomas were found to be in the young and middle age group, with extended duration of symptoms, and most had > 5cm sized lesions with intermediate grade and high-grade disease. This in general is the same prototype clinicopathologic characteristics of STS reported worldwide [3, 6, 11, 14]. A further review of the treatment modalities revealed that slightly less than a fifth of our patient population had the benefits of either neoadjuvant chemotherapy and or radiotherapy. In comparison to previous publications, a larger proportion of our patients had primary surgical management [6]. This, in part, may be due to the predominance of truncal, rather than extremity, lesions in our series when comparing the anatomic locations with other studies [6, 21, 22]. The significance of the site in determining the use of neoadjuvant treatment (particularly radiotherapy) is that except in some histologic subtypes (embryonal rhabdomyosarcoma, synovial sarcoma, Ewing’s sarcoma) found responsive to neoadjuvant chemoradiation, the risk of toxicity in delivering maximal radiotherapy dose to the truncal/ visceral STS exclusive of contiguous organs and neuro-osseo-vascular structure [6, 23-27] is high. Of equal importance is the varied and inconsistent response to chemotherapy noted between different histologic subtypes along with chemotoxicity [28-32]. These factors make surgery the gold standard; albeit more so in anatomic locations that precludes the use of chemoradiation. The adjuvant treatment options available in our practice setting, a sub-Sahara African developing country, were very limited compared to the standards of care in other parts of the world like the Asian-Pacific region [6]. We believe this is due to the challenges of instituting early radiotherapy in our setting which may be due to competing volume from other commoner radiosensitive malignancies.

Liposarcoma, leiomyosarcoma and malignant fibrous histiocytoma were the predominant subtypes of STS in our review. Although the same pattern was noted in other reports subtle differences in proportional ranking exist across continental boundaries [6, 22, 33]. In the period under review about two-third of our patients had surgical treatment but only about a fifth received oncoplastic reconstruction to cover the wide defect created after extirpative surgery. This suggests that a viable reconstructive unit enhances the ability to resect hitherto unresectable lesions. Closely related to this is our positive margin rate which is directly proportional to the size and proximity to vital structures with the culprit in our case being mostly anatomic constraints. While adjudged not ideal, it is however acceptable to leave planned microscopic positive surgical margins, after weighing the risk of recurrence and morbidity of radical surgery along with full discussion with the patient [3, 34]. A positive resection margins is an independent prognostic factor for local recurrence [8, 35], but since neither a positive surgical margin with or without local recurrence adversely affects overall survival [36], then substantial functional compromise or amputation of a limb may not be a worthwhile endeavor. Other reasons that possibly contributed to unresectability include late/delayed presentation, inability to pay out of pocket for treatment and sometimes unwillingness on the part of the patient or caregiver to undergo surgery.

Truncal and visceral STS (GIST and retroperitoneal STS) have a worse prognosis [37] than extremity lesions mainly because local recurrence in the former can lead to mortality whereas it does not in the latter except in the setting of metastases [38]. Local recurrence may be a reflection of tumor biology and the acknowledged pivotal role of neoadjuvant radiotherapy in reducing local recurrence [39]. Our post- operative morbidity/mortality rate and duration of hospital admission compared favorably with other, more advanced centers [11, 40, 41], despite not matching their operative volume.

Over a third of our patients were attending the surgical out-patient’s clinics 20 months after surgery. The reasons for loss of many to long term follow-up are not known, thus the survival pattern of this cohort might never be determined. A local recurrence rate of 17% and distant metastases of 11% at eighteen months post-surgery in the setting of little or no adjuvant care suggest adequacy of surgical care as it compares with findings by other authors [3, 6, 22].

The epidemiology of GIST is completely unknown [42] and it is suggested that it constitutes a sixth of all STS; our review however showed a lower percentage which is not inclusive of autopsy and incidental finding by other authors [42, 43]. We found most cases of GIST in our series to be of gastric origin, which is similar to findings in North America and Asia [44, 45]. GIST have a high tendency to seed with florid peritoneal sarcomatosis noted in our patients who could not have resection at laparotomy, en-bloc resection was achievable in those with contiguous organ involvement; usual culprit being the spleen and tail of the pancreas.

Anthracycline and alkylating agents have been our first line chemotherapy over the years of review in view of the non-availability of newer agents like the monoclonal antibodies (olaratumab, pembolizumab, sunnitumab), eribulin, aldoxorubicin and trabectidin. Beyond this, is the fact that histology staining techniques/molecular diagnosis which unravels the heterogeneity and guides the escalation to 2^nd^ or 3^rd^ line regimens are expensive and available strictly only in the research or investigative settings [46].

About 750 cases of adult malignancies are seen per *anum* in this center. The age standardized ratio for STS in males is 0.8/100,000 while that in females is 0.5/100,000 [47]. This is similar to the global prevalence of STS of 1% seen in literature review [1-4].

## Conclusion

There is scarcity of concise report on STS in sub-Saharan Africa hence the importance of assessing and auditing its outcome in a developing country like Nigeria. The low local recurrence rate and distant metastasis following surgical resection despite little or no adjuvant therapy emphasizes the importance of surgical care and negative resection margins in a LMIC like ours. There can be improvement in the reported outcomes if patients present with early stage disease. Secondly, noted gaps in our surgical practice such as absence of intra-operative frozen section, multi-disciplinary tumor (MDT) board for STS and absence of a proper follow up protocol to be able to find out survival patterns needs to be inculcated at the institutional level to monitor and evaluate quality of care.

### What is known about this topic

- STS is a rare group of neoplasm of mesenchymal tissue whose management is surgical resection with neo or adjuvant chemo radiation.

### What this study adds

- Sarcomas represent less than a percent of cases of malignancies seen in our practice with most patients presenting late. The predominant histologic type is liposarcoma while the trunk is the commonest site and high grade histologic type predominates;
- Surgery remains the main modality of care with little chemoradiation option. The recurrence rate and distance metastases are less than 20%;
- Follow-up is poor with majority being lost to follow-up by 36 months post-surgical intervention.
